# Effect on Syndecan-1 and Hyaluronan Levels Depending on Multiple Organ Failure, Coagulopathy and Survival: An Observational Study in Major Trauma Patients

**DOI:** 10.3390/jcm13226768

**Published:** 2024-11-10

**Authors:** Mareen Braunstein, Thorsten Annecke, Kathrin Frey, Thomas Kusmenkov, Markus Wörnle, Ludwig Ney, Wolfgang Böcker, Viktoria Bogner-Flatz

**Affiliations:** 1 Department of Orthopaedics and Trauma Surgery, Musculoskeletal University Centre Munich (MUM), University Hospital, LMU Munich, Ziemssenstraße 5, 80336 Munich, Germany; kathrinfrey@outlook.de (K.F.); wolfgang.boecker@med.uni-muenchen.de (W.B.); viktoria.bogner@med.uni-muenchen.de (V.B.-F.); 2 Department of Anaesthesiology and Critical Care Medicine, Merheim Medical Centre, University of Witten/Herdecke, 51109 Cologne, Germany; annecket@kliniken-koeln.de; 3 Niels-Stensen-Klinken, Marienhospital Osnabrück, Bischofsstr. 1, 49072 Osnabrück, Germany; thomas.kusmenkov@niels-stensen-kliniken.de; 4 Emergency Department, University Hospital, LMU Munich, Ziemssenstraße 5, 80336 Munich, Germany; markus.woernle@med.uni-muenchen.de; 5 Department of Anesthesiology, University Hospital, LMU Munich, Ziemssenstraße 5, 80336 Munich, Germany; ludwig.ney@med.uni-muenchen.de

**Keywords:** major trauma, glycocalyx shedding, syndecan-1, hyaluronan, multiple organ failure, coagulopathy

## Abstract

**Background:** Major trauma, as well as traumatic hemorrhagic shock go along with early damage to the endothelial glycocalyx (EG). Shed glycocalyx constituents can activate the innate immune system and aggravate secondary injury. Subsequently, we investigated the relationship between glycocalyx shedding and the occurrence of coagulopathy, multiple organ failure (MOF) and outcome in our cohort after severe trauma. **Methods:** We included multiple trauma patients, as defined by Injury Severity Score (ISS). Polytraumatized patients must have arrived in our level 1 trauma center within 60 min after trauma. Retrospectively, patients were assigned to predefined clinical conditions, based on injury severity (ISS ≥ 16 points), multiple organ failure (MOF score ≥ 6 points), need for massive transfusion (≥10 RBC units/first 24 h), coagulopathy (prothrombin time < 70% at 0 h) and survival (90-day survival). Syndecan-1 (Sdc-1) and hyaluronan (HA) plasma concentrations were evaluated immediately (0 h), 6 h and 12 h after trauma. **Results:** 49 patients (mean ISS 35.7 ± 12.1 SD, mean age 45.78 ± 15.6 SD) were included in this study. A total of 37 patients (75.5%) survived, while 12 patients died within the observation period of 90 days after trauma (24.5%). A total of 77% of all patients suffered multiple organ failure (MOF score ≥ 6, n = 30). Initial prothrombin time at 0 h was <70% in 31 patients. Plasma concentrations of circulating both glycocalyx constituents showed a significant increase over the first 12 h after trauma (*p* = 0.001; *p* = 0.008). Patients with multiple organ failure showed significantly increased hyaluronan concentrations at all three time points (*p* = 0.007/0.006/<0.001), and the syndecan-1 levels were significantly elevated 12 h after trauma in the MOF group (*p* = 0.01). Patients with coagulopathy on admission exhibited significantly higher hyaluronan levels at 12 h (*p* = 0.042). Non-survivors showed significantly increased syndecan-1 levels at 12 h after trauma (*p* = 0.024). **Conclusions:** Glycocalyx shedding occurs immediately after major trauma. Coagulopathy is associated with significantly increased plasma hyaluronan. Further, significant changes in plasma concentrations within the first 12 h help to identify subgroups at risk for developing MOF and death.

## 1. Introduction

Despite injury prevention programs and novel treatment strategies like “damage control resuscitation”, trauma remains a relevant health issue worldwide [1].

Severe traumatic hemorrhage and hemorrhagic shock are still one of the leading causes of mortality in multiple-trauma patients [2]. When focusing on traumatic hemorrhage, class I hemorrhage (defined as volume loss of up to 15% of total blood volume) does not regularly affect the endothelial surface layer, whereas different hemorrhagic shock states are capable of interrupting glycocalyx integrity [3]. In experimental studies, severe hemorrhagic shock resulted in endothelial injury even before resuscitation, and glycocalyx shedding was able to mediate and worsen coagulopathy [4]. Therefore, the management of traumatic bleeding and trauma-induced coagulopathy (TIC) are in the focus of primary trauma resuscitation—especially in the very early post-traumatic phase. This is especially true, as the initial trauma is known to trigger coagulofibrinolytic modulation, which can lead to an increased bleeding. In these cases, hemostasis becomes even more difficult to control. Trauma-induced coagulopathy is a complex and multifactorial phenomenon occurring in one third of all multiple trauma patients. TIC is demonstrated to be an independent risk factor for trauma mortality [5]. Its pathogenesis involves multiple factors, with traumatic endotheliopathy being implicated in the onset of coagulopathy, as noted in earlier studies [6,7]. The breakdown of the endothelial surface layer compromises its barrier function, which leads to increased capillary permeability. Further, this exposure of e.g., heparan sulfate from endothelial cells due to EG shedding plays a role in coagulopathy through so called auto-heparinization. The loss of the glycocalyx integrity further reveals the endothelial cell surface, which encourages the adhesion of platelets and thereby triggers thrombin formation [8]. Nonetheless, the connection between circulating levels of glycocalyx components such as syndecan-1 or hyaluronan and the full range of pathophysiological coagulation in the early stage of trauma remains insufficiently explored. An observational study in trauma patients could recently show that elevated circulating levels of syndecan-1 were linked to sustained and heightened activation of coagulation, disorder of anticoagulation mechanisms, enhanced fibrinolytic activity, and consumption coagulopathy in 48 patients after trauma [9].

Trauma and shock-related release of this soluble EG components contribute to auto-heparinization, as previously mentioned, and seem to further augment inflammatory reactions by positive feedback mechanisms [10]. This status of very early damage of the endothelial glycocalyx layer after injury is called the “endotheliopathy of trauma” [11]. Increased plasma concentrations of syndecan-1, heparan sulfate and hyaluronan are accepted markers to detect this “glycocalyx shedding”. A specific characterization of traumatic hemorrhagic shock is the accompanying extensive tissue damage, which leads to a very early release of danger-associated molecular patterns (DAMPs) [12]. To address this pronounced traumatic tissue damage, a range of swift innate immune responses are triggered to restore the so-called “pre-injury state” [13]. The previously mentioned damage-associated molecular patterns (DAMPs), including, e.g., ATP and HMGB-1, can convey their signals to polymorphonuclear leukocytes through pattern-recognition receptors (PRRs) such as toll-like receptors (TLRs) [14]. Xiao et al. characterized a phenomenon termed “genetic storm”, along with a trauma-related reassessment of leukocytes after experiencing a severe injury. This response even appears to vary individually based on the specific pattern of injury [15,16].

Basically, the innate immune system was found to closely interact with the endothelium, as described by Aird and colleagues [17]. The endothelial surface layer is a dynamic construct with several important functions [18], such as cellular signaling, neutralizing reactive oxygen species (ROS) to reduce oxidative damage to the endothelium and preserving a homeostatic environment, to mention only some of these important functions [19]. If the EG is damaged, the deterioration of these functions might be the reason for further pathophysiologic derangements. Following trauma and significant blood loss, the shedding of endothelial glycocalyx compounds enhances the pro-inflammatory response by facilitating the binding of inflammatory cytokines. Furthermore, reactive oxygen species and pro-inflammatory cytokines such as TNF-α and IL-6 activate endothelial cells, which further amplify innate immune responses.

Transferring these insights on EG derangement to a trauma patient, the initial insult seems to promote primary injury to the EGL and has been reported to aggravate the post-traumatic systemic cytokine activation and ROS generation triggered by ischemic/reperfusion injury. Recent studies in multiple-trauma patients indicate that the severity of injury or hemorrhage was associated with higher circulation levels of glycocalyx components, especially syndecan-1 and hyaluronan. Consequently, both could serve as “shedding markers” in trauma patients [9,20].

From our point of view, glycocalyx integrity might become one of the key facts to consider in trauma management—and it has not been adequately addressed in trauma management concepts up to date. This might mostly be due to the fact that the role of the endothelium and especially its dynamic surface layer is still not completely understood in major trauma patients. To underline the importance of endotheliopathy in polytraumatized patients, a recent systematic review proposed that future trauma resuscitation could or even should focus on the glycocalyx—to minimize the endothelial disruption and address its prompt reconstruction. The traumatic impact and its associated morbidity might thereby be attenuated [19]. Considering the patient’s initial “endothelial glycocalyx status” could allow for a more personalized and targeted approach to trauma resuscitation, potentially improving outcomes, especially in the very early post-traumatic phase. The recognition of patients at risk is still a relevant challenge, and maybe a better insight into endothelial degradation can help to further improve the chances of survival and recovery for trauma patients. Therefore, it remains of ongoing importance to investigate this vulnerable cohort of multiple-trauma patients and to evaluate the relationship between the immediate early release (0 h) of syndecan-1 and hyaluronan from the endothelial glycocalyx into the circulation and the occurrence of traumatic coagulopathy, multiple organ dysfunction and early trauma death.

This current observational study was planned to evaluate the circulating plasma concentrations of syndecan-1 and hyaluronan, as well as their early post-traumatic dynamic over the first 12 h after trauma—starting immediately on admission (0 h time point). This 0 h time point should serve as a relatively “native” analysis of EG degradation/shedding without the potential impact of intensive trauma resuscitation. In a second step, we intended to correlate these plasma levels to different patient conditions with distinct clinical outcome parameters.

## 2. Materials and Methods

### 2.1. Patients and Blood Sampling

The Regional Ethical Review Board of Ludwig-Maximilians-University Munich, Germany (reference number 344-11), approved this observational study. Our investigation was performed following Good Clinical Practice in the Munich University Clinic, LMU, which is a level 1 trauma center. Patients (aged 18 years and older) with major trauma (ISS ≥ 16 points) were included in our study if they were admitted to our clinic`s emergency department within 1 h after trauma. All included patients or a legal representative signed an informed consent statement. Patients who did not survive the first 24 h after trauma were excluded. All patients were treated according to the Advanced Trauma Life Support guidelines. After trauma resuscitation and/or urgent intervention/operation, patients were admitted to the intensive care unit. Several pre-conditions effecting immune response or coagulation were defined as exclusion criteria, e.g., acute infectious disease, immunosuppressive therapy, terminal disease, vascular disease or pre-existing coagulopathy. For each patient, the following personal and medical records were collected: demographic data, clinical parameters and co-morbidities, days on the intensive care unit and the length of hospital stay [21,22].

Patients were retrospectively related to different clinical conditions depending on the following parameters: injury severity, massive blood transfusion, coagulopathy, development of multiple organ failure and 90-day survival. Injury severity was assessed using the Injury Severity Score (ISS) according to AIS98. The groups were divided according to the median value into two sub-groups (ISS ≤ 34 and ISS > 34). Multiple organ failure (MOF) was described using the MOF score modified by Lefering and colleagues [23]. A MOF score ≥ 6 (the median value of our cohort) was assumed as multiple organ failure. Massive transfusion was characterized when a patient received ≥ red blood cell (RBC) units in the first 24 h after trauma. Traumatic coagulopathy was defined as 0 h prothrombin time of less than 70%. Survival was defined as 90-day survival after trauma [24].

### 2.2. Blood Sampling

We collected blood samples immediately on admission, which is called the 0 h time point. The subsequent blood samples were drawn 6 h and 12 h after the traumatic event. All samples were stored at −80 °C until further analyses were performed, as described in the following section [25].

### 2.3. Plasma Concentration

The concentration of syndecan-1 protein was assessed using the Human sCD138 (syndecan-1) ELISA Kit from Diaclone SAS (Product Number: 950.640; Diaclone SAS, 6 Rue Docteur Jean-François-Xavier Girod, BP 1985, 25020 Besancon Cedex, France). In accordance with the kit protocol, plasma samples, along with standard, control and blank series, were added to the wells of the microtiter plate. Subsequently, 50 µL of biotinylated anti-CD138 antibody was introduced into each well, and the plate was incubated for one hour at 37°. After incubation, the supernatant was discarded, and the wells were washed three times with 300 µL of wash buffer. Following this, 100 µL of streptavidin-HRP solution was added, and the plate was incubated for an additional 30 min. A further washing step was then performed. To trigger the color reaction, 100 µL of TMB (3,3′,5,5′-Tetramethylbenzidine) substrate solution was added to each well, and the plate was incubated in the dark at room temperature for 12 to 15 min. The reaction was stopped by adding 100 µL of H_2_SO_4_ solution to each well. The absorbance was then measured photometrically at 450 nm, with the signal being directly proportional to the syndecan-1 concentration [24].

To determine the concentration of hyaluronan, the Hyaluronan Enzyme-Linked Immunosorbent Assay Kit (HA-ELISA) from Biosciences Inc. was utilized (Product Number: K-1200; Echelon Biosciences Inc., 675 Arapeen Drive, Suite 302, Salt Lake City, UT 84108, USA). Following the provided protocol, standard series, blank controls, zero controls and plasma samples were added to the incubation plate. Next, 50 µL of hyaluronic acid (HA) detector was pipetted into each well, excluding the blank controls. After gentle mixing, the plate was incubated at 37 °C for one hour. Subsequently, 100 µL from each well was transferred to the detection plate, followed by another incubation at 30 °C for four minutes. The wells were then emptied and washed three times with 200 µL of wash buffer. Afterward, 100 µL of enzyme solution was added to each well, and the plate was incubated for 30 min at 37 °C. After this incubation, the wells were washed again as described. To each well, 100 µL of substrate solution containing p-nitrophenyl phosphate was added, and the plate was incubated in the dark at room temperature. After 15 min, the first photometric reading was taken at 405 nm. The intensity of the color reaction was inversely proportional to the hyaluronan concentration. The total incubation time was determined by monitoring the zero controls and the 1600 ng/mL standard. When the optical density ratio (ODZERO/OD1600) exceeded 3.0, the reaction was terminated by adding 50 µL of stop solution [24].

### 2.4. Statistical Analyses

The statistical analysis was performed using SPSS Statistics Version 26.0 (IBM Corporation; Armonk, NY, USA). For repeated measures analyses, we selected a statistical model (repeated measures MANOVA, SPSS General Linear Model) to consider sequential measurements and to analyze protein dynamics. The statistical model included the following factors: time to indicate significant protein dynamics, injury severity assessed by ISS, development of multiple organ failure detected by MOF score ≥ 6 points, coagulopathy expressed by prothrombin time (<70% at 0 h), massive transfusion (≥10 units/first 24 h) and 90-day survival. If the findings were statistically significant, the single time points were again statistically analyzed using non-parametric Mann–Whitney U test (Greenhouse–Geisser correction) [24].

## 3. Results

### 3.1. Clinical Data

**Figure 1:**
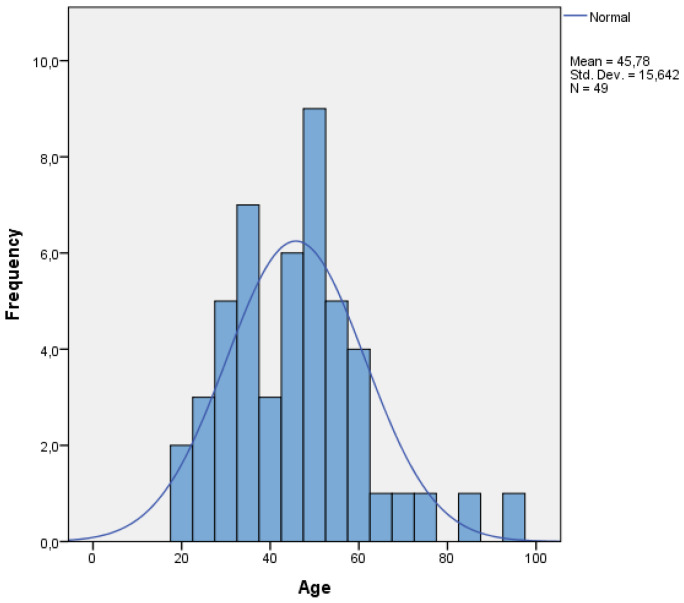
Age distribution of all patients (n = 49).

A total of 49 patients were included in this study. The mean age was 45.7 years (±15.6 SD; age showed normal distribution using Kolmogorov–Smirnov test; see Figure 1). A total of 35% of all patients were female (n = 17). A total of 76% survived the traumatic event (90-day survival; survivors n = 37; non-survivors n = 12). The mean ISS was 35.7 points (±12.1 SD; ISS ≤ 34: n = 27 and ISS > 34: n = 22). The median MOF score was 6. Multiple organ failure developed in 77% of the patients (MOF < 6: n = 19; MOF ≥ 6: n = 30). A total of 43% of patients had a massive transfusion within the first 24 h after the trauma (survivors n = 10; non-survivors n = 11). Coagulopathy occurred in 31 patients (63%, survivors n = 21, non-survivors n = 10). Table 1 shows the detailed clinical data of all included patients.

### 3.2. Plasma Concentrations in the Early Post-Traumatic Period

**Figure 2:**
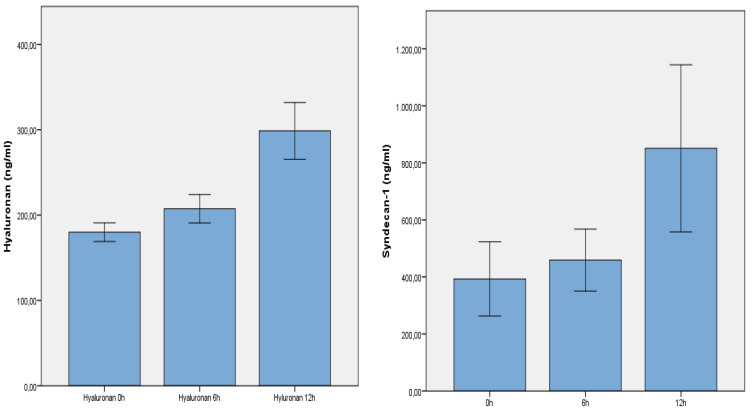
It shows the significant increase of plasma hyaluronan and plasma syndecan-1 after trauma in all patients (linear regression model; Greenhouse–Geisser correction).

The shedding of syndecan-1 and hyaluronan showed a significant dynamic in all patients. Both components of the endothelial glycocalyx (hyaluronan/syndecan-1) showed a significant concentration dynamic in all patients within the first 12 h after trauma (*p* = 0.001/0.008) (Figure 2).

All plasma concentration levels were given in ng/mL. Bars represent mean values, with error bars representing +/− 1SE. A MANOVA analysis for repeated measurements was used.

### 3.3. Plasma Concentrations and Multiple Organ Failure

**Figure 3:**
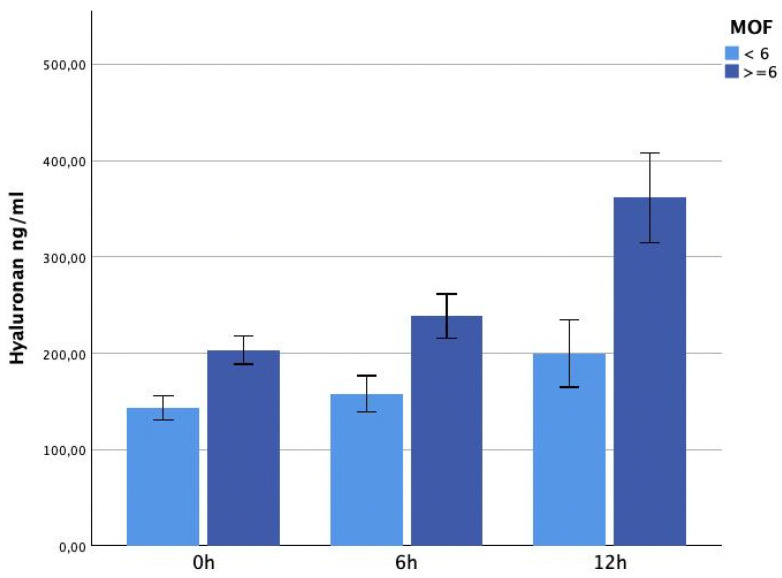
It shows hyaluronan plasma levels within 12 h after trauma in patients presenting with MOF. Hyaluronan concentrations were significantly higher in patients with MOF on admission 0 h (*p* = 0.007), after 6 h (*p* = 0.006) and after 12 h (*p* < 0.001).

The hyaluronan concentrations were already significantly higher immediately on admission (*p*= 0.007) and again 6 h (*p* = 0.006) and 12 h (*p* < 0.001) after trauma in the MOF-group (Figure 3).

**Figure 4:**
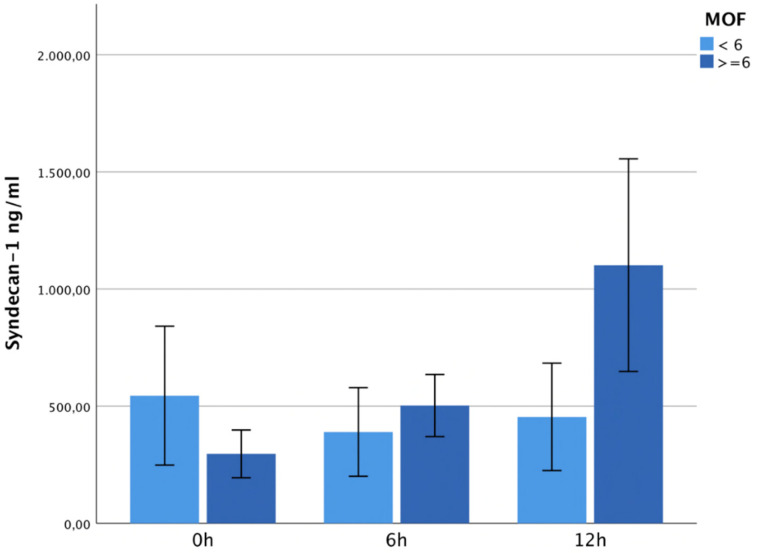
It shows syndecan-1 plasma levels within 12 h after trauma depending on MOF. The level of syndecan-1 was not significantly increased on admission and 6 h after trauma but increased five-fold 12 h after trauma in the MOF group, showing a significant difference compared to the non-MOF patients (*p* = 0.01).

The level of syndecan-1 was not significantly increased on admission and 6 h after trauma in patients developing MOF, but later increased five-fold in the MOF group, showing a significant difference compared to the non-MOF patients at 12 h after trauma (*p* = 0.01). See Figure 4.

### 3.4. Plasma Concentrations and Coagulopathy

**Figure 5:**
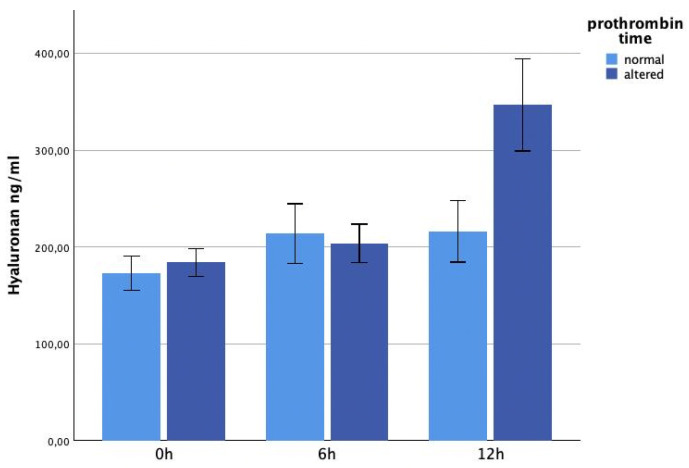
It shows hyaluronan plasma levels within 12 h after trauma, correlated with coagulopathy. Patients with coagulopathy (measured 0 h after trauma) showed significantly higher hyaluronan levels at 12 h (*p* = 0.042).

**Figure 6:**
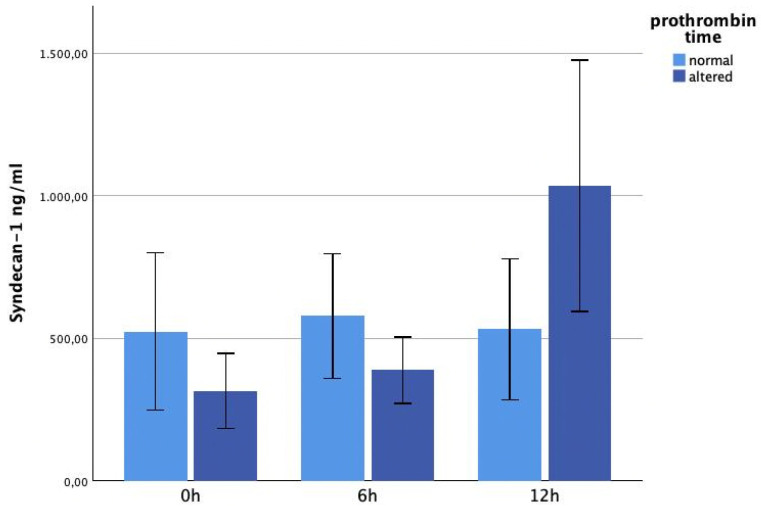
It shows syndecan-1 plasma levels within 12 h after trauma, correlated with coagulopathy. There were no significant differences between the two groups.

The glycocalyx markers did not show significant differences on admission, depending on immediate coagulopathy. The hyaluronan levels were significantly higher in the coagulopathy group at 12 h (*p* = 0.042). See Figure 5. The syndecan-1 concentrations were higher in patients developing coagulopathy, but this increase was not significant (Figure 6).

### 3.5. Plasma Concentrations and Massive Transfusion

**Figure 7:**
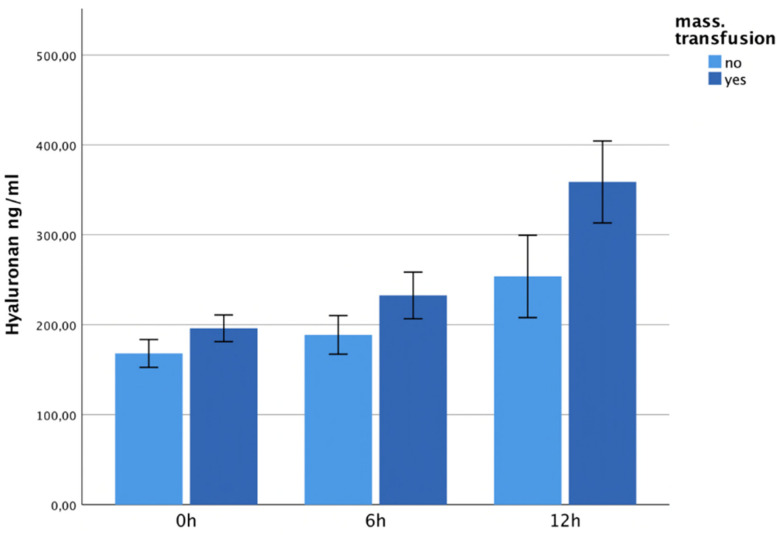
It shows hyaluronan plasma levels within 12 h after trauma, correlated with massive transfusion. The patients in the massive transfusion group showed significantly increased hyaluronan levels 12 h after the traumatic event (*p* = 0.006).

**Figure 8:**
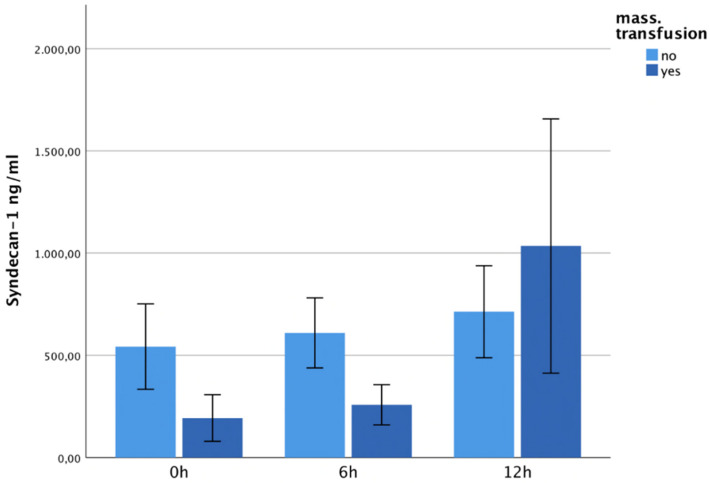
It shows syndecan-1 plasma levels within 12 h after trauma, correlated with massive transfusion. The patients with massive transfusion showed higher syndecan-1 levels 12 h after trauma, but these differences did not reveal statistical significance.

All multiple-trauma patients who received a massive transfusion (>10RBC/24 h after trauma) showed significantly higher hyaluronan levels than those without massive transfusion (12 h; *p* = 0.006). See Figure 7. The patients with massive transfusion also showed higher syndecan-1 levels at 12 h after trauma, but these differences did not reveal statistical significance (Figure 8).

### 3.6. Plasma Concentrations and Outcome

**Figure 9:**
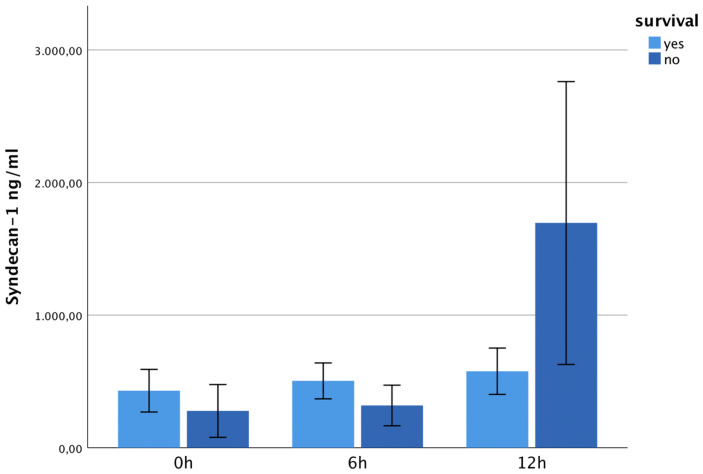
It shows syndecan-1 plasma levels within 12 h after trauma, correlated with the outcome. The syndecan-1 levels showed a significant maximum in non-survivors at 12 h post-trauma (*p* = 0.024).

**Figure 10:**
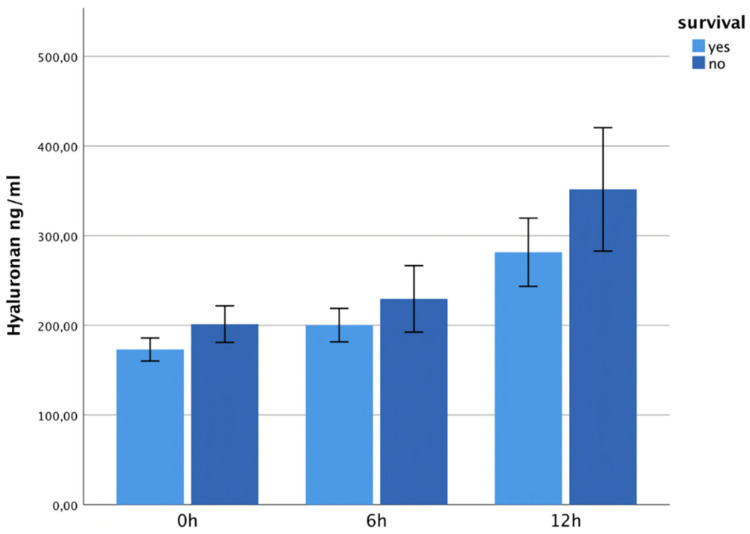
It shows hyaluronan plasma levels within 12 h after trauma, correlated with the outcome. The hyaluronan levels were non-significantly higher in survivors at 0 h, 6 h and 12 h.

The syndecan-1 levels were higher in survivors at 0 h and 6 h and showed a significant maximum in non-survivors at 12 h post-trauma (*p* = 0.024). See Figure 9. The hyaluronan concentrations showed no statistically significant difference between survivors and non-survivors at 0 h, 6 h or 12 h (Figure 10).

## 4. Discussion

Trauma-induced glycocalyx shedding goes along with numerous modifications, including intensified systemic inflammation, augmented vascular permeability and impairment of coagulation. These post-traumatic pathophysiological changes aggravated the existing derangements of the injured patients. Consequently, a better understanding and insight into EG shedding and its implication on the post-traumatic clinical course of our trauma patients remains crucial.

We hereby present a study in major trauma patients investigating circulating glycocalyx components (syndecan-1 and hyaluronan) as markers of glycocalyx shedding at three different time points in the very early post-traumatic period.

Significantly increased hyaluronan concentrations were detected in patients with multiple organ failure during the whole observation period, indicating that the immediate early release of hyaluronan (within 60 min) can help predict patients with subsequent MOF, as already defined by an MOF score above 6 on admission. Syndecan-1 plasma levels significantly indicated mortality in the very early post-traumatic period, as non-survivors showed significantly increased syndecan-1 levels at 12 h after trauma. Patients with coagulopathy already present on admission exhibited significantly higher hyaluronan levels at 12 h. These findings are in line with precedent publications on EG integrity in cases of major trauma. In this context, Johansson and colleagues prospectively investigated a major trauma cohort and detected a significant correlation between injury severity and syndecan-1 levels [27]. In contrast, we could not detect this significant correlation in our cohort. However, the mean ISS values in both studies differ significantly, showing more severely injured patients in our study (mean ISS 17 vs. 34 points). Furthermore, we harshly defined the time of the first blood samples collection as “no later than 1 h after trauma” and standardized the follow-up blood sampling to the traumatic event. Our data suggests that the timing of the measurement could be crucial for its future use as a marker. At 12 h, non-survivors exhibit significantly higher syndecan-1 levels, which is most likely due to a persistent state of shock. This perfectly aligns with our finding that syndecan-1 is related to the extent of MOF after major trauma. The patients who develop critical multiple organ failure have higher syndecan-1 levels at 12 h. The correlation of raised syndecan-1 levels with unfavorable or fatal clinical developments is not only “trauma specific” as this phenomenon could also be detected in patients after cardiac arrest and return of spontaneous circulation as part of the “post-resuscitation syndrome” and in a sepsis cohort [28].

In an earlier study conducted by our group, we were able to demonstrate that patients who underwent resuscitation due to cardiac arrest and subsequently achieved return of spontaneous circulation exhibited marked endothelial glycocalyx (EG) shedding immediately following ROSC [24]. Further, the immediate increase of syncdecan-1 and hyaluronan significantly correlated with organ failure and survival after the return of circulation, as measured immediately after successful resuscitation. Current evidence suggests that endothelial dysfunction and glycocalyx damage are critical contributors to ischemia/reperfusion injury and the early onset of systemic inflammatory response syndrome (SIRS). The endothelial glycocalyx is essential for regulating key processes, such as leukocyte–endothelial interactions, preserving vascular barrier integrity and mediating flow-induced shear stress transmission [29,30]. In this regard, Halbgebauer et al. evaluated in their cohort of polytrauma patients that the extent of traumatic endotheliopathy significantly correlated with the degree of glycocalyx shedding. [31]. Rodriguez et al. could show in their large cohort study of more than 400 patients that the syndecan-1 levels of the non-survivors were significantly elevated when compared to the survivors. The authors summarized that syndecan-1 might be used as an “index of endotheliopathy of trauma” [32].

Glykokalyx shedding and especially syndecan-1 release has been postulated to initiate auto-heparinization in other investigations before [7]. The latter is a fatal mechanism in bleeding patients and leads to raised morbidity and mortality after trauma. In contrast to other investigations and against our primary hypothesis, syndecan-1 levels initially after trauma (0 h) do not differentiate patients with pathological prothrombin time in the early post-trauma period [6]. In addition, these findings do not support the assumption of direct early auto-heparinization by high levels of circulating EG compounds. However, patients with initial pathological prothrombin time and massive transfusion exhibit higher syndecan-1 levels in the 12 h post-trauma time course as a trend. We hypothesize that this may be caused by a prolonged state of shock and coagulopathy in this subgroup. It has been shown that the latter is an independent risk factor for increased mortality rates in polytrauma patients [33], closely linked to a higher snydecan-1 release due to tissue injury, complement activation and mortality [31]. Besides these indistinct results for syndecan-1, hyaluronan plasma levels significantly correlate with the prothrombin time and massive transfusion.

Hyaluronan and syndecan-1 significantly correlate with multiple organ failure in our cohort. In this context, Puskarich et al. demonstrated similar connections in patients with severe sepsis, depending on the extent of MOF or in patients after an OHCA and resuscitation [28]. Hyaluronan is not only a marker of glycocalyx shedding but is also considered a pro-inflammatory mediator that indicates systemic inflammation [34].

The strength of our study, compared to previous ones, is the early timing of the initial blood draws, which were strictly standardized to the traumatic event. The first blood sampling time point was performed on admission, within 1 h after trauma; the second at 6 h; and the third at 12 h, all standardized to the traumatic event. Other studies on syndecan-1 in trauma described admission as the first blood sampling time point, with a median of 75 min (mean or median) after trauma or earlier, but without follow-up after 6 and 12 h. We thereby might be able to close this gap in knowledge by the timed management of blood sampling in our study.

Another strength, and the most distinct difference of our study compared to the three other existing clinical investigations, is the focus on injury severity. The median ISS in two studies was 17 points and 21 points in the other. In contrast, the median ISS in our study is 34 points, representing a cohort of severely injured patients [6,7,27]. This may explain why we cannot entirely confirm the findings of the precedent results of other investigations. The fact of having included a more severe and morbid study cohort is reflected in the higher syndecan-1 levels, as compared to other studies. Our mean syndecan-1 levels on admission are 6-fold higher than those reported in other studies. We may speculate that the reported predictive character in syndecan-1 level in other clinical investigations may be attenuated among a cohort of more severely injured patients, such as ours.

For a deeper understanding of the pathomechanisms of endotheliopathy following trauma, animal models can be particularly beneficial. Hemorrhagic shock models, especially, have shown that major bleeding causes distinctive shedding of the endothelial glycocalyx in the postcapillary venules of rat skeletal muscle and mesentery [35]. Regarding severe blood loss in experimental settings, another group observed that glycocalyx shedding does not occur uniformly across all vascular beds. The most significant shedding was observed in the endothelium of the pulmonary and intestinal vasculature. The EGL in these locations simultaneously showed the highest levels of reactive oxygen species generation [4]. Furthermore, other animal models could demonstrate that the presence of polytrauma with hemorrhagic shock resulted in different vascular barrier alterations concerning permeability and shedding patterns when compared to hemorrhagic shock alone. This recent experimental study could show that non-traumatic hemorrhagic shock displayed glycocalyx degradation without any disruption of the vascular barrier in rats [36]. In summary, the ESL and its complex interactions are still not fully elucidated in trauma settings, and both animal models and clinical investigations are needed to implement a deeper understanding into this complex field of trauma research.

This current investigation was able to answer some relevant questions in the context of traumatic endotheliopathy, but further research is still needed. The rise in plasma concentrations of glycocalyx components can significantly impact the clinical outcomes for patients by indicating the extent of endothelial damage and dysfunction. We were able to show that elevated levels of these EG compounds were associated with worse clinical outcomes due to the underlying pathophysiological changes that were described in detail before. Monitoring these plasma levels can thus serve as a valuable marker to guide therapeutic interventions and thereby potentially improve patient management in critical care settings. In this context, a more detailed examination of different subgroups, such as elderly patients, injury patterns or fracture locations, would be desirable, as the influence of these factors on trauma outcomes appears to be significant [37,38].

Our study group`s further research directions must focus on a larger sample size of polytrauma patients. We are already planning a follow-up study with a larger cohort and a longer observation period to strengthen the statistical power of our study, allowing us to “generalize” our results. This new study will need to be restructured, as we intend to measure additional inflammatory biomarkers and other glycocalyx components, such as heparan sulfate and biglycan, to gain a broad insight into the extensive role of EGL in cases of trauma. In this context, hyaluronan and syndecan-1 levels have been shown to correlate with other inflammatory markers, but their specific association with endothelial damage sets them apart, providing a more direct reflection of glycocalyx integrity and endothelial health from our point of view. In summary, hyaluronan and syndecan-1 concentrations provide important insights into the “endothelial status” and thereby the severity of post-traumatic changes in trauma patients. Therefore, we can conclude that shedding parameters can complement, rather than replace, other biomarkers. Regarding these facts, the combined use of several markers can enhance the overall assessment of patient status and might guide clinical decision-making in the future.

## 5. Limitations

From our perspective, the relatively small sample size is the main limitation of our presented work. The limited sample size is due to the single-center approach, the study design, complex logistics and targeted patient selection.

A further limitation might be that the changes in circulating hyaluronic acid concentration could be affected by the treatment with fresh-frozen plasma (FFP) in our population of severely injured trauma patients. Consequently, further research and analyses by our group are currently underway to (1) investigate the effect of FFP administration on syndecan-1 and hyaluronan levels and (2) correlate the effect to distinct clinical outcome parameters. However, in this context, current evidence is controversial, and a recent experimental animal study from 2024, for example, has not found any evidence for higher circulating HA levels following FFP treatment [39].

In this current work, we were not able to show other plasma inflammatory cytokine and chemical mediator levels such as IL-6 or HMGB1, although these are known to be related to the pathophysiology of trauma. In this specific setting, we focused solely on glycocalyx components. Further investigation should focus on a longitudinal observation of endothelial shedding markers and cytokine changes. Particularly, changes in the glycocalyx components and cytokine levels might be able to reflect the progression or resolution of trauma-induced post-traumatic disorder. For instance, in conditions such as acute respiratory distress syndrome (ARDS), more frequent monitoring of inflammatory and endothelial disorders via glycocalyx components and cytokines could provide valuable insights into the efficacy of therapeutic strategies and might help to guide subsequent treatment decisions.

Taking all the prior considerations into account, we remain confident that our findings are highly significant, holding great potential and encouraging further research, as we were already able to outline upcoming follow-up projects—as mentioned above—that have emerged from the promising initial results of this study.

## 6. Conclusions

Regarding the results of this investigation, we were able to present immediate 0 h plasma levels of free circulating glycocalyx constituents in a cohort of severely polytraumatized patients. Additionally, we could demonstrate a short-term follow-up in multiple-trauma patients by subsequent serial measurements in the very early post-traumatic phase of 12 h after the traumatic event.

Our results could depict a significant concentration dynamic of hyaluronan and syndecan-1 very early after trauma. Further, our results could impressively identify an association between the extent of post-traumatic glycocalyx shedding and MOF, massive transfusion, coagulopathy and adverse outcome. In detail, polytrauma leads to significant syndecan-1 glycocalyx shedding, which is associated with post-traumatic outcomes and MOF, already representing a potential biomarker 12 h after the trauma.

Even earlier, hyaluronan shedding could indicate worse outcomes following major trauma in patients who present with coagulopathy or MOF as it was already significantly higher in these cohorts.

## Figures and Tables

**Table 1:** Clinical data, including age, sex and injury severity, assessed by ISS, 90-day survival and cause of death (n = 49) [26].

	AGE	SEX	ISS	90-Day Survival	Cause of Death
1	38	M	33	no	MOF
2	59	M	50	no	MOF
3	74	W	26	no	MOF
4	44	M	57		
5	46	M	34		
6	34	M	54		
7	43	M	41		
8	58	M	29		
9	48	W	50	no	TBI
10	25	W	50		
11	51	W	45	no	TBI
12	57	M	57		
13	40	M	29		
14	71	W	20	no	MOF
15	32	M	34		
16	51	W	43	no	TBI
17	49	M	24	no	MOF
18	32	M	34	no	MOF
19	28	W	34		
20	33	M	34		
21	37	M	42		
22	48	W	35		
23	29	M	41		
24	51	M	29		
25	61	W	34		
26	35	M	57	no	MOF
27	36	M	29		
28	65	W	27		
29	35	W	36		
30	84	W	29		
31	94	M	59	no	Respiratory failure
32	28	W	45		
33	37	W	41	no	TBI
34	20	M	22		
35	53	M	36		
36	50	M	29		
37	46	M	22		
38	53	M	22		
39	25	W	16		
40	38	W	18		
41	21	M	22		
42	52	M	20		
43	46	M	34		
44	60	M	38		
45	24	W	50		
46	53	M	27		
47	54	M	50		
48	44	M	34		
49	51	M	45		

## Data Availability

Data is contained within the article.

## Abbreviations

The following abbreviations are used in this manuscript:

EG	Endothelial glycocalyx
ISS	Injury Severity Score
MOF	Multiple organ failure
RBC	Red blood cells
Sdc-1	Syndecan-1 hyaluronan
HA	Hyaluronan
DAMPs	Danger associated molecular patterns
ATP	Adenosine triphosphate
HMGB-1	High mobility group box-1
PRRs	Pattern-recognition receptors
TLR	Toll-like receptor
EGL	Endothelial glycocalyx layer
ROS	Reactive oxygen species
ROSC	Return of spontaneous circulation
